# Circulating tumour cells at baseline and late phase of treatment provide prognostic value in breast cancer

**DOI:** 10.1038/s41598-021-92876-8

**Published:** 2021-06-29

**Authors:** Shuyun Pang, Hanjun Li, Shu Xu, Liying Feng, Xueping Ma, Yanan Chu, Bingjie Zou, Shaohua Wang, Guohua Zhou

**Affiliations:** 1 Department of Clinical Pharmacy, State Key Laboratory of Analytical Chemistry for Life Science and Jiangsu Key Laboratory of Molecular Medicine, Jinling Hospital, Medical School of Nanjing University, Nanjing, 210002 China; 2 Department of General Surgery, Jinling Hospital, Medical School of Nanjing University, Nanjing, 210002 China; 3 School of Pharmaceutical Science, Southern Medical University, Guangzhou, 510515 China; 4 School of Basic Medical Science and Clinical Pharmacy, China Pharmaceutical University, Nanjing, 210000 China

**Keywords:** Prognostic markers, Cancer

## Abstract

To determine the prognostic value of the timing of circulating breast tumour cell measurement during treatment, peripheral blood from 164 patients with breast disease was collected. Circulating tumour cells (CTCs) were enriched by using immunomagnetic nanospheres (IMNs) and were identified by using immunofluorescent staining. The CTC shows nuclear-positive, EpCAM-positive, CK19-positive, and CD45-negative. Patients with CTC positivity (> 19/7.5 mL blood) had shorter progression-free survival (PFS) and overall survival (OS) than those with negative results (≤ 19/7.5 mL blood) at baseline. Surgery caused an increase in the number and prevalence of CTCs, and the effect disappeared on day 14 after surgery. During adjuvant chemotherapy, CTCs detected before therapy was only correlated with PFS; however, CTCs at the end of adjuvant chemotherapy were correlated with both PFS and OS. The PFS and OS of the CTC-positive group were significantly shorter than those of the CTC-negative group at the end-point follow-up visit. The prognostic value of CTCs at different measurement time points was demonstrated during breast cancer treatment. Surgery and chemotherapy affected the prevalence of CTCs, leading to different prognostic relevance of CTCs at different treatment stages. CTCs detected at baseline or in the late phase of treatment are preferable for prognosis.

## Introduction

Circulating tumour cells (CTCs) are released from tumours into the vascular system. CTCs show characteristics of tumour and stem cell capability^1,2^, which is related to metastasis. Increasing evidence has shown that CTCs are the seeds of tumour metastasis^2–5^. Therefore, CTCs are potential biomarkers for diagnosis ^6^ and prognosis^7,8^ of tumours. In addition, CTC detection is non-invasive, enabling monitoring of tumour progression^9^ and timely evaluation of efficacy^3^.

CTCs as biomarkers were first used in the prognosis of metastatic breast cancer^7^. Many prospective studies including large-scale multi-centre research confirmed that the number of CTCs over 5/7.5 ml blood detected by CellSearch system in metastatic breast cancer was associated with poor prognosis^7,10–12^. Moreover, CTCs have been incorporated into the 7th edition of the American Joint Committee on Cancer staging system for breast cancer as an index of distant metastases^13^. The number of CTCs detected by CellSearch system over 1/7.5 ml blood was also confirmed to be prognostic for progression-free survival (PFS) and overall survival (OS) in patients with non-metastatic breast cancer^14^.

However, many studies on the prognostic relevance of CTCs in breast cancer have used the number of CTCs at one time point before or after a certain therapy such as surgery^15^ and chemotherapy^16^ for correlation analysis. In fact, the number of CTCs varies during treatment, and the therapy could affect the number of CTCs. For example, the number of CTCs increased during the following 3–4 days after surgery^17^. One study showed that PFS and OS were significantly associated with the number of CTCs after adjuvant chemotherapy but not at 1-week post-surgery^18^, indicating that the measurement time point could affect the prognostic value of CTCs. Therefore, it is necessary to study the prognostic value of the timing of CTC detection during the treatment of breast cancer. Although some studies have compared the prognostic relevance of CTCs in breast cancer before and after treatment^8,19,20^, few studies have included different treatment stages.

On the other hand, the capture efficiency of the CTC enrichment method also affects its prognostic accuracy. Although epitope-based CTC enrichment methods (such as CellSearch system) may miss some CTCs that do not express the corresponding antigens, they are still the most commonly used CTC enrichment methods because of the simple and convenient operation. Therefore, when using epitope-based CTC enrichment method to study the prognostic value of CTC, it is necessary to choose a CTC enrichment method with high capture efficiency, so as to avoid further loss of CTC. The capture efficiency of epitope-based CTC enrichment method is mainly affected by the surface to volume ratio of the capture medium. Magnetic nanoparticles have a relatively high surface to volume ratio, inducing the high binding ability and CTC capture efficiency. However, the magnetic response of magnetic nanoparticles is not fast enough, and they suffer higher loss rate. Immunomagnetic nanospheres (IMNs) with quick magnetic response overcome this defect of magnetic nanoparticles^21^. IMNs are made of Pst-AAm-COOH as the core, and coated with 5 layers of nano-γ-Fe_2_O_3_ to get a large magnetic saturation value^21^, so that the IMNs could be captured within 1 min with commercial magnetic medium^21^. Therefore, the whole progress of CTC enrichment could be completed within 5 min with ~ 94% recovery rate^21^.

Therefore, by using IMNs to capture the CTC, the prognostic relevance of CTCs at the time point of pre-surgery, pre-adjuvant chemotherapy, final adjuvant chemotherapy, and perioperative period was demonstrated to clarify which time points the CTC has prognostic value.

## Results

### Optimization of CTC enrichment method and threshold demarcation

Quick-response IMNs can capture tumour cells in blood with an efficiency of more than 94% after only 5 min of incubation^21^. Therefore, it is a rapid and sensitive CTC detection method. However, the previously described method recommends using 1.5 mL of blood to detect CTCs, which is less than the sample volume (7.5 mL) in the CellSearch system. Because a larger volume of blood could increase the sensitivity of CTC detection, we increased the blood sample volume to 7.5 mL for the IMN-based CTC detection.

To accurately and efficiently enrich CTCs in a 7.5 mL blood sample, we optimised the capture conditions by performing experiments with different concentrations of IMNs and incubation times, and then compared the capture recovery rate of each condition. The recovery rate was calculated by dividing the number of captured MCF-7 cells by the number of input MCF-7 cells. As shown in Fig. S1a, 0.13 mg/mL IMNs gave a 97.1% recovery rate, which was higher than that of 0.07 mg/mL IMNs (85.7% recovery rate) and 0.20 mg/mL IMNs (91.4% recovery rate). Incubation times of 5 min and 10 min showed recovery rates of over 95% (Fig. S1b), but 1 min and 15 min incubation times showed lower recovery rates. Therefore, 0.13 mg/mL IMNs and 5-min incubation were used in subsequent experiments.

To investigate the capture specificity of IMNs, three kinds of epithelial tumour cells (HCT116, A549, and MCF-7) and leukocytes were added to PBS (7.5 mL) and captured by IMNs. The recovery rates of all epithelial tumour cells were over 92%, but the recovery rate of leukocytes was 16.6% (Fig. S1c). The non-specific capture of leukocytes may be caused by the non-specific absorption of IMNs. Therefore, we further increased the washing time, and non-specific adsorption was reduced by washing multiple times (Fig. S1d). In addition, leukocytes could be distinguished by CD45 staining, thus, it did not affect CTC counts. Leukocyte residues are inevitable. But it is no significant difference in the number of leukocytes between the breast cancer and healthy donors (Fig. S1e).

Anti-EpCAM antibody also captures epithelial non-tumour cells in the blood. Thus cells displaying nuclear-positive, CK19-positive, and CD45-negative by immunocytochemistry, were also observed in the blood samples from healthy donors (Fig. S1f). Blood samples from 19 healthy donors were collected and tested for CTC-positive threshold demarcation, and the results showed that the mean epithelial cell count was 8.89, and the standard deviation (SD) was 3.72. Therefore, the threshold of the CTC-positive sample was 19, calculated by adding the mean epithelial cell counts and three times the SD.

**Figure 1:**
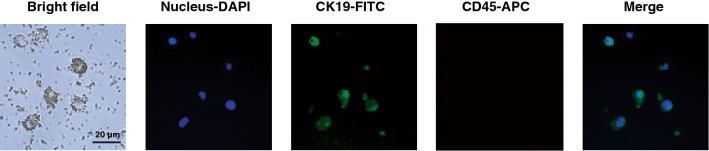
Images of IMN-captured and immunofluorescence-stained CTCs from a patient peripheral blood specimen.

CTCs were detected in the peripheral blood of the patients. CTCs were wrapped in magnetic beads and were nuclear-positive, EpCAM-positive, CK19-positive, and CD45-negative (Figs. 1 and S3). Interestingly, we found that CTCs were also detected in 42.3% of the patients who were diagnosed with benign breast diseases (Figure S1f). This might be related to the presence of epithelial cells in the blood of different patients due to personal condition or medication, indicating that CTCs are not a good biomarker for diagnosing benign breast diseases.

### Patient characteristics and CTC prevalence at baseline

Between 2015 and 2016, 138 patients with breast cancer were enrolled in the study. CTC detection was successfully performed in 110 patients (79.7%) before a new line of treatment, except for 28 patients (20.3%), whose blood samples were not qualified or the information was not collected (Fig. S2). The CTCs enumeration results are summarized in Table S1. The characteristics of patients with CTC detection results at baseline are summarised in Table 1. Of the 110 patients, 55 (50.0%) were CTC-negative (≤ 19/7.5 mL blood) and 55 (50.0%) were CTC-positive (> 19/7.5 mL blood). The age range of patients was 23–84 years, and the average age of the patients was 52.7. Eleven patients (10.0%) had ductal carcinoma in situ, 88 patients (80.0%) had invasive ductal carcinoma, and 11 patients (10.0%) had other pathological types of breast cancer, including neuroendocrine carcinoma, invasive lobular carcinoma, metaplastic carcinoma, and mixed carcinoma. CTCs were associated with distant metastasis (*P* = 0.0429). Patients with distant metastasis had a higher prevalence of CTCs, which is in line with the status quo. Most metastatic breast cancers have a higher CTC prevalence than non-metastatic breast cancers in different studies^22–25^. There were no significant differences in intrinsic subtype, stage, risk categories, tumour size, lymph node status, oestrogen receptor (ER) status, progesterone receptor (PR) status, human epidermal growth factor receptor 2 (HER2) status, and Ki67 status between the CTC-positive and CTC-negative groups of patients (*P* > 0.05).

**Table 1:** Patient characteristics and prevalence of CTCs.

	All patients (N = 110)	Baseline CTC negative (N = 55)	Baseline CTC positive (N = 55)	*P* value
Age	52.7 (23–84)	53.6 (34–84)	51.7 (23–79)	0.3545^a^
**Histology**				0.1482^c^
Ductal carcinoma in situ	11	3 (27.2%)	8 (72.8%)	
Invasive ductal carcinoma	88	48 (54.5%)	40 (45.5%)	
Other types	11	4 (36.4%)	7 (63.6%)	
**Intrinsic subtype**				0.4874^c^
Luminal A-like	25	11 (44.0%)	14 (56.0%)	
Luminal B-like (HER2 negative)	32	13 (40.6%)	19 (59.4%)	
Luminal B-like (HER2 positive)	24	15 (62.5%)	9 (37.5%)	
Erb-B2 overexpression	19	10 (52.6%)	9 (47.4%)	
Basal-like	10	6 (60.0%)	4 (40.0%)	
**Stage**				0.2702^b^
0	8	2 (25.0%)	6 (75.0%)	
I	19	11 (57.9%)	8 (42.1%)	
II	38	25 (65.8%)	13 (34.2%)	
III	18	8 (44.4%)	10 (55.6%)	
IV	20	6 (30.0%)	14 (70.0%)	
N/A	7			
**Risk categories**				0.4010^b^
Low risk	8	2 (25.0%)	6 (75.0%)	
Intermediate risk	62	39 (62.9%)	23 (37.1%)	
High risk	34	13 (38.2%)	21 (61.8%)	
N/A	6			
**Tumour (TNM staging)**				0.5570^b^
0	7	2 (28.6%)	5 (71.4%)	
1	37	19 (51.4%)	18 (48.6%)	
2	55	31 (56.4%)	24 (43.6%)	
3	7	1 (14.3%)	6 (85.7%)	
4	2	2 (100%)	0 (0%)	
N/A	2			
**Node (TNM staging)**				0.1483^b^
0	53	29 (54.7%)	24 (45.3%)	
1	16	11 (68.7%)	5 (31.3%)	
2	15	6 (40.0%)	9 (60.0%)	
3	12	4 (33.3%)	8 (66.7%)	
N/A	14			
**Metastasis (TNM staging)**				0.0429^c^
0	89	49 (55.1%)	40 (44.9%)	
1	20	6 (30.0%)	14 (70.0%)	
N/A	1			
**ER**				0.8352^c^
Negative	33	17 (51.5%)	16 (48.5%)	
Positive	77	38 (49.4%)	39 (50.6%)	
**PR**				0.8472^c^
Negative	63	31 (49.2%)	32 (50.8%)	
Positive	47	24 (51.1%)	23 (48.9%)	
**Her-2**				0.3286^c^
Negative	67	31 (46.3%)	36 (53.7%)	
Positive	43	24 (55.8%)	19 (44.2%)	
**Ki67**				0.5541^c^
≤ 20%	41	19 (46.3%)	22 (53.7%)	
> 20%	69	36 (52.2%)	33 (47.8%)	

^a^*P* value from Mann–Whitney test.

^b^*P* value from Pearson’s chi-squared test for trend.

^c^*P* value from Pearson’s chi-squared test.

### Prognostic relevance of CTC level at baseline

**Figure 2:**
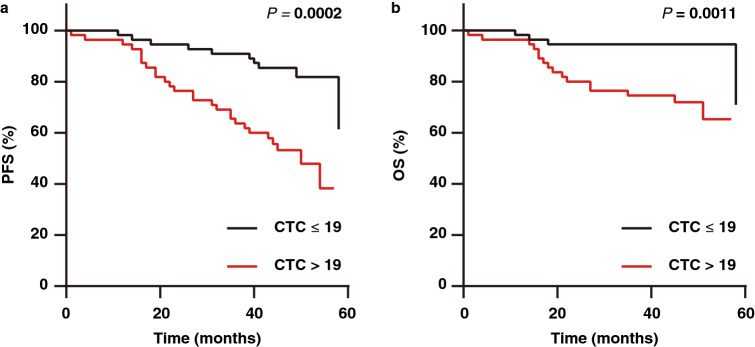
Kaplan–Meier curves showing estimated PFS (**a**) and OS (**b**) for patients with baseline CTC negativity (CTC ≤ 19) and CTC positivity (CTC > 19).

Survival data were obtained for the 110 patients available for evaluation. The median follow-up duration was 45 months. A total of 37 patients (33.6%) developed disease recurrence, and 20 (18.2%) died during follow-up. Fifty-five patients (50.0%) were CTC-positive compared with 55 patients (50.0%) were CTC-negative. Patients with CTC positivity had a significantly shorter time to progression than the patients with CTC negativity (*P* = 0.0002; hazard ratio [HR], 3.56; 95% confidence interval [CI], 1.86–6.82), and patients with CTC positivity had a significantly shorter time to death than patients with CTC negativity (*P* = 0.0011; HR, 4.98; 95% CI, 2.06–12.02) (Fig. 2a and b). These results indicated that the presence of CTCs at baseline was associated with an unfavourable prognosis.

Various clinicopathological prognostic factors, including baseline CTCs, intrinsic subtype, histology, stage, risk categories, tumour size, lymph node status, visceral metastases, ER status, PR status, HER2 status, and Ki67 status were considered for the univariate Cox regression analysis (Table 2). Baseline CTCs (*P* = 0.000; HR, 3.93; 95% CI, 1.84–8.39), stage (*P* = 0.000; HR, 2.17; 95% CI, 1.57–3.00), and risk categories (*P* = 0.000; HR, 3.68; 95% CI 1.94–7.00) were significantly correlated. CTC positivity at baseline, high stage, and high-risk categories signified a shorter PFS. In Cox multivariate analysis, baseline CTCs (*P* = 0.002; HR, 3.63; 95% CI, 1.60–8.27) and stage (*P* = 0.031; HR 1.75; 95% CI, 1.05–2.90) were significantly correlated (Table 2), indicating that both baseline CTCs and stage were independent factors for PFS. For OS, baseline CTCs (*P* = 0.003; HR, 6.50; 95% CI, 1.89–22.36), stage (*P* = 0.000; HR, 4.22; 95% CI, 2.32–7.68), risk categories (*P* = 0.000; HR, 8.99; 95% CI 3.04–26.57), and ER status (*P* = 0.049; HR, 0.41; 95% CI, 0.17–1.00) were related to poor prognosis. Similarly, baseline CTCs (*P* = 0.042; HR, 4.35; 95% CI, 1.05–17.98) and stage (*P* = 0.003; HR, 3.79; 95% CI, 1.57–9.14) were independent factors for OS in multivariate analysis.

**Table 2:** Cox univariate analysis and multivariate analysis of CTCs at baseline.

	PFS				OS			
	Cox univariate analysis		Cox multivariate analysis		Cox univariate analysis		Cox multivariate analysis	
	HR (95% CI)	*P* value	HR (95% CI)	*P* value	HR (95% CI)	*P* value	HR (95% CI)	*P* value
CTC at baseline	3.93 (1.84–8.39)	0.000	3.63 (1.60–8.27)	0.002	6.50 (1.89–22.36)	0.003	4.35 (1.05–17.98)	0.042
Intrinsic subtype	1.19 (0.91–1.55)	0.197	1.00 (0.61–1.66)	0.986	1.24 (0.87–1.77)	0.235	0.92 (0.40–2.14)	0.855
Stage	2.17 (1.57–3.00)	0.000	1.75 (1.05–2.90)	0.031	4.22 (2.32–7.68)	0.000	3.79 (1.57–9.14)	0.003
Risk categories	3.68 (1.94–7.00)	0.000	1.66 (0.62–4.44)	0.309	8.99 (3.04–26.57)	0.000	2.07 (0.48–8.96)	0.333
Histology	1.59 (0.76–3.33)	0.218	1.81 (0.72–4.57)	0.210	1.69 (0.63–4.52)	0.299	3.27 (0.64–16.81)	0.156
ER status	0.52 (0.27–1.01)	0.054	0.43 (0.14–1.32)	0.139	0.41 (0.17–1.00)	0.049	0.17 (0.02–1.33)	0.091
PR status	0.40 (0.19–0.86)	0.019	0.47 (0.20–1.12)	0.089	0.46 (0.17–1.28)	0.136	0.53 (0.14–1.97)	0.343
HER2 status	1.07 (0.55–2.08)	0.833	0.78 (0.34–1.79)	0.561	1.06 (0.44–2.57)	0.900	0.37 (0.10–1.41)	0.146
Ki67 status	1.97 (0.90–4.33)	0.091	1.16 (0.46–2.92)	0.760	3.06 (0.90–10.46)	0.074	2.02 (0.36–11.41)	0.426
Final model				0.000				0.000

### CTCs in the perioperative period and their prognostic relevance

Surgery can affect the number of CTCs in a patient’s blood^17^. We investigated the CTCs during the perioperative period. The number of CTCs significantly increased on the first day after surgery (*P* = 0.0001) (Fig. S4a), and the CTC prevalence increased from 44.6% to 65.8% (*P* = 0.0073). The prevalence increase persisted until 7 d after surgery (*P* = 0.0147), and the number of CTCs levelled (*P* = 0.0634) (Fig. S4b). There was no significant difference between the number of CTCs 14 d after surgery and the number of CTCs before surgery (*P* = 0.3718), and the CTC prevalence decreased to 54.2% at 14 d after surgery (*P* = 0.2899) (Fig. S4c). Therefore, CTC count varied in the perioperative period. It is necessary to further investigate the prognostic value of CTCs at different measurement time points during the perioperative period.

**Figure 3:**
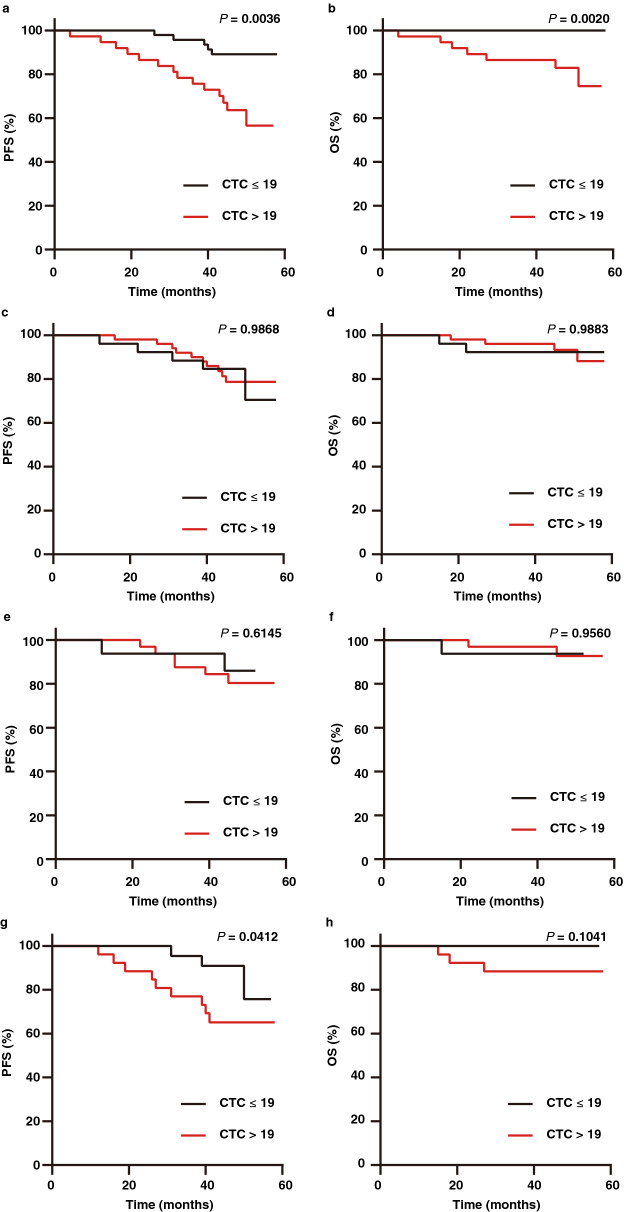
Kaplan–Meier curves showing estimated PFS and OS for patients with CTC negativity (CTC ≤ 19) and CTC positivity (CTC > 19) before surgery (**a**, **b**), 1 d after surgery (**c**, **d**), 7 d after surgery (**e**, **f**) and 14 d after surgery (**g**, **h**).

As Fig. 3 shows, the PFS (*P* = 0.0036; HR, 4.05; 95% CI, 1.62–10.10) and OS (*P* = 0.0020; HR, 10.71; 95% CI, 2.39–48.06) of preoperatively CTC-positive patients were significantly shorter than those of CTC-negative patients. However, there was no significant difference in PFS (*P*_1 d_ = 0.9868; HR_1 d_, 0.99; 95% CI_1 d_, 0.34–2.90; *P*_7 d_ = 0.6145; HR_7 d_, 1.50; 95% CI_7 d_, 0.35–6.53) and OS (*P*_1 d_ = 0.9883; HR_1 d_, 0.99; 95% CI_1 d_, 0.18–5.41; *P*_7 d_ = 0.9560; HR_7 d_, 0.93; 95% CI_7 d_, 0.08–10.61) between CTC-positive and CTC-negative patients on days 1 and 7 after surgery. However, CTCs detected 14 d after surgery were related to PFS (*P* = 0.0412; HR, 2.74; 95% CI, 0.88–8.55), indicating that the variation of CTC counts caused by surgery could affect the prognostic relevance of CTCs.

### Prognostic relevance of CTCs before initial and final adjuvant chemotherapy

**Figure 4:**
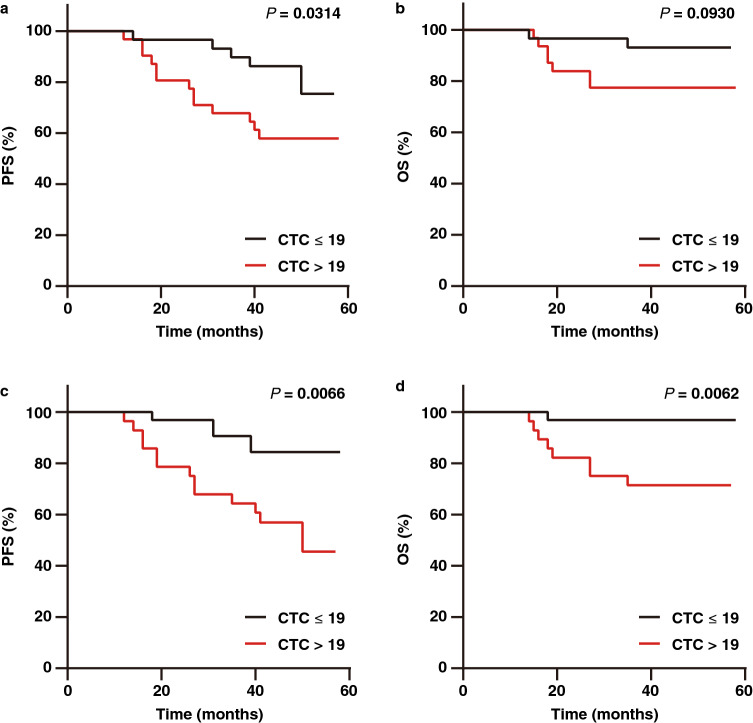
Kaplan–Meier curves showing estimated PFS and OS for patients with CTC negativity (CTC ≤ 19) and CTC positivity (CTC > 19) before initial chemotherapy (**a**, **b**) and before final chemotherapy (**c**, **d**).

After surgery, many patients received adjuvant chemotherapy. CTCs in the blood samples from 60 patients were measured before initial adjuvant chemotherapy and before the final adjuvant chemotherapy to investigate the effect of adjuvant chemotherapy on the prognostic relevance of CTCs. The prevalence of CTCs before adjuvant chemotherapy (51.7%) was higher than that after adjuvant chemotherapy (46.7%), indicating that chemotherapy had an impact on CTCs. The PFS of CTC-positive patients before the first chemotherapy was significantly shorter than that of CTC-negative patients (*P* = 0.0314; HR, 2.92; 95% CI, 1.16–7.37) (Fig. 4a), but there was no significant difference in OS (*P* = 0.0930; HR, 3.51; 95% CI, 0.95–12.97) (Fig. 4b). After adjuvant chemotherapy, CTC-positive patients had significantly shorter PFS (*P* = 0.0066; HR, 3.48; 95% CI, 1.47–9.59) and OS (*P* = 0.0062; HR 10.27; 95% CI, 2.75–38.31) than CTC-negative patients (Fig. 4c and d), indicating that CTCs after adjuvant chemotherapy are more associated with prognosis. Therefore, CTCs in the late phase of disease treatment have prognostic value.

### Prognostic relevance of CTC at the end-point follow-up visit

**Figure 5:**
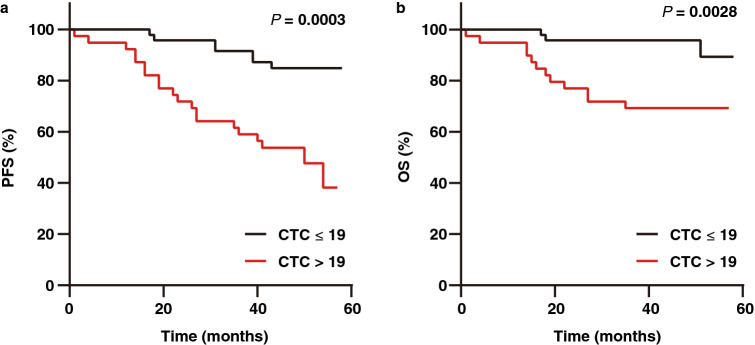
Kaplan–Meier curves showing estimated PFS (**a**) and OS (**b**) for patients with CTC positivity (CTC > 19) and CTC negativity (CTC ≤ 19) at the end-point follow-up visit.

To investigate whether CTCs during the later phase of disease treatment are associated with prognosis, the prognostic relevance of CTCs at the end-point follow-up visit in 86 patients was analysed. There were 39 CTC-positive cases (45.3%) and 47 CTC-negative cases (54.7%). Twenty CTC-positive patients (51.3%) developed disease recurrence, while disease recurrence occurred in only 7 CTC-negative patients (14.9%). Twelve CTC-positive patients (30.8%) died, while death only occurred in 3 CTC-negative patients (6.4%). CTC-positive cases showed a significantly shorter PFS (*P* = 0.0003; HR, 4.25; 95% CI, 1.96–9.09) and OS (*P* = 0.0028; HR, 5.52; 95% CI, 1.98–15.37) than CTC-negative cases (Fig. 5a and b). Therefore, CTCs at the end-point follow-up visit were associated with prognosis.

In univariable cox regression analysis, CTCs at the end-point-follow-up visit (*P* = 0.001; HR, 4.21; 95% CI, 1.77–9.99), stage (*P* = 0.000; HR, 2.55; 95% CI, 1.70–3.83), risk categories (*P* = 0.000; HR, 4.65; 95% CI 2.12–10.19) and PR status (*P* = 0.007; HR, 0.26; 95% CI 0.10–0.69) were the significate factor of PFS (Table 3). In Cox multivariate analysis, CTCs at the end-point-follow-up visit (*P* = 0.010; HR, 3.51; 95% CI, 1.35–9.13) and PR status (*P* = 0.023; HR 0.27; 95% CI, 0.09–0.84) were significantly correlated (Table 3), indicating that CTCs at the end-point-follow-up visit was the independent factor for PFS. For OS, CTCs at the end-point-follow-up visit (*P* = 0.009; HR, 5.39; 95% CI, 1.52–19.11), stage (*P* = 0.000; HR, 5.22; 95% CI, 2.53–10.78) and risk categories (*P* = 0.001; HR, 28.78; 95% CI 3.81–217.68) were related to unfavourable prognosis (Table 3). However, CTCs at the end-point-follow-up visit wasn’t the independent factor for OS in multivariable models with clinical covariates (Table 3).

**Table 3:** Cox univariate analysis and multivariate analysis of CTCs at the end-point follow-up visit.

	PFS				OS			
	Cox univariate analysis		Cox multivariate analysis		Cox univariate analysis		Cox multivariate analysis	
	HR (95% CI)	*P* value	HR (95% CI)	*P* value	HR (95% CI)	*P* value	HR (95% CI)	*P* value
CTC at the end-point follow-up visit	4.21 (1.77–9.99)	0.001	3.51 (1.35–9.13)	0.010	5.39 (1.52–19.11)	0.009	3.02 (0.70–13.05)	0.138
Intrinsic subtype	1.30 (0.97–1.74)	0.084	0.82 (0.44–1.51)	0.517	1.30 (0.88–1.93)	0.193	0.72 (0.26–1.94)	0.511
Stage	2.55 (1.70–3.83)	0.000	1.52 (0.63–3.68)	0.354	5.22 (2.53–10.78)	0.000	3.38 (0.68–16.73)	0.135
Risk categories	4.65 (2.12–10.19)	0.000	2.40 (0.49–11.60)	0.278	28.78 (3.80–217.68)	0.001	7.03 (0.37–133.32)	0.194
Histology	1.25 (0.52–3.00)	0.615	2.09 (0.69–6.31)	0.191	2.00 (0.66–5.99)	0.218	10.48 (1.72–63.67)	0.011
ER status	0.50 (0.23–1.10)	0.086	0.40 (0.08–1.86)	0.240	0.46 (0.16–1.26)	0.130	0.13 (0.01–1.51)	0.104
PR status	0.26 (0.10–0.69)	0.007	0.27 (0.09–0.84)	0.023	0.48 (0.15–1.51)	0.208	0.41 (0.08–2.09)	0.285
HER2 status	1.12 (0.52–2.39)	0.777	0.69 (0.26–1.80)	0.444	1.16 (0.42–3.20)	0.773	0.50 (0.10–2.51)	0.399
Ki67 status	2.06 (0.83–5.11)	0.120	1.24 (0.40–3.82)	0.709	3.71 (0.84–16.50)	0.085	1.09 (0.18–6.64)	0.926
Final model				0.000				0.000

## Discussion

CTCs have been proven associated with the prognosis of breast cancer. However, therapy can affect CTCs, especially surgery^15^. It has been reported that the number of CTCs increases 3–4 days after surgery^17^. Our data showed that the number and prevalence of CTCs increased at 1 d and 7 d after surgery. No significant differences in PFS and OS were observed between days 1 and 7 after surgery for CTC-positive and CTC-negative patients, but CTCs measured on day 14 after surgery were related to PFS. Therefore, the measurement time point of CTCs affects their prognostic value.

CTCs at baseline showed prognostic relevance in many reports^26–28^ as well as in the present study, because most CTCs at baseline were measured before any new line of treatment thus the effects of therapy were limited. However, some patients missed the CTC measurement time point at baseline, therefore if these patients want to benefit from the prognosis of CTCs, another CTC-measurement time point, at which the number of CTCs has prognostic value, should be determined.

Therefore, we investigated the prognostic relevance of CTCs before and after adjuvant chemotherapy in addition to the perioperative period. The prevalence of CTCs before adjuvant chemotherapy (51.7%) was higher than that after adjuvant chemotherapy (46.7%), indicating that chemotherapy had an impact on CTCs. However, CTCs after chemotherapy showed more associations with PFS and OS than those before adjuvant chemotherapy, revealing that a CTC-measurement time point closer to the later phase of the treatment had a more obvious prognostic value. To validate this hypothesis, the prognostic relevance of CTCs at the end-point follow-up visit was investigated. Although the end-point follow-up visit did not occur after a fixed period, CTCs at the end-point follow-up visit showed a prognostic value. Therefore, in addition to the baseline, CTCs at the late phase of the disease or at the end of treatment also have prognostic value.

The research of the present study has some limitations: (1) It was a single-centre prospective study and the number of patients enrolled was limited. Patient stratification analysis did not proceed because of the limited number of patients. The mobility of patients resulted in a smaller sample size at follow-up visit and the treatment monitoring; (2) Although the performance of IMNs for CTC collection was validated, this method was not compared to other platforms such as CellSearch system; (3) More treatment stages, such as neoadjuvant chemotherapy, should be considered for inclusion in a study to assess the prognostic value of CTCs measured at different time points.

## Conclusion

In this prospective trial, the prognostic value of the timing of CTC measurement for breast cancer in the Chinese population was demonstrated. CTCs change dynamically during treatment, which can affect prognosis. Surgery led to an increase in the number and prevalence of CTCs on the first day after surgery and did not return to the preoperative level until 14 d after surgery. The CTC prevalence at the baseline and end-point follow-up visits was related to PFS and OS, while the CTCs detected before chemotherapy were only related to PFS. The CTCs detected at the last adjuvant chemotherapy were more correlated with prognosis than those before adjuvant chemotherapy in the analysis of paired samples. Therefore, CTCs at baseline or in the late phase of treatment are preferable for prognosis.

## Materials and methods

### Patients and study design

Between 2015 and 2016, 164 patients with breast disease who were diagnosed or treated at Jinling Hospital were included in our study. Peripheral blood was collected before surgery and recorded as a baseline then when available before each treatment until the end of the adjuvant chemotherapy. A discard tube was used to collect the first 1 mL of bloods to avoid epithelial skin cells contamination. Progression or non-progression was evaluated according to clinical practice based on clinical and radiological evaluations. Peripheral blood specimens from 19 healthy donors were used as negative controls.

The study was approved by the Committee on Biomedical Research Ethics of Jinling Hospital (2015NZGKJ-082) and was performed in accordance with the Declaration of Helsinki. All participants provided written informed consent.

### Cell culture

MCF-7 (HTB-22., ATCC, VA, USA), HCT-116 (CCL-247., ATCC), and A549 (CCL-158., ATCC) were grown in a 25-cm^2^ flask containing high-glucose Dulbecco’s modified Eagle’s medium (DMEM) (Gibco, NY, USA) supplemented with 10% fetal bovine serum (FBS) (Gibco) under 5% CO_2_ at 37 °C.

### Capture of spiked tumour cells in mock samples

MCF-7 cells (passage14) were placed in phosphate-buffered saline (PBS) as mock samples. CTCs were captured by quick-response immunomagnetic nanosphere (IMN)-modified anti-epithelial-cell-adhesion-molecule (EpCAM) monoclonal antibody (Wuhan Jiayuan Quantum Dots Corporation, Ltd., Wuhan, China)^21^. IMNs were added to 7.5 mL of sample, and the mixture was incubated for 5 min at 37 °C. After magnetic separation, the captured cells were fixed with 4% paraformaldehyde (Nanjing Chemical Reagent Co., Ltd., Nanjing, China), permeabilized, blocked with 0.1% Triton-X 100 (Sigma-Aldrich, MO, USA) and 1% bovine serum albumin (BSA) (Amresco, OH, USA), and stained with 30 μg/mL 4',6-diamidino-2-phenylindole (DAPI) (Sigma-Aldrich), FITC-labeled anti-keratin-19 (CK19) monoclonal antibody (Abcam, MA, USA), and APC-labelled anti-protein-tyrosine-phosphatase-receptor-type-C (CD45) monoclonal antibody (Abcam). After washing, the captured cells were placed in a cell culture dish (Greiner, Kremsmünster, Austria) for fluorescence microscopy imaging. CTCs were identified as cells that had round to oval morphology, were positive for DAPI and CK19, and negative for CD45. All processes are completed within 2 h.

### Detection of CTCs in breast disease patients and healthy donors

Eight millilitres of blood was drawn into EDTAVacutainer tubes (BD, NJ, USA). The blood samples were stored at 4 °C and processed within two h after collection. Seven point five millilitres of blood was incubated with IMNs for 5 min. After enrichment, the subsequent process is the same as the process described in “Capture of spiked tumour cells in mock samples” section.

### Statistical analysis

Statistical analyses were performed using IBM SPSS Statistics (version 24.0, IBM, Armonk, NY, USA). Pearson’s chi-square test was used to compare the categorical variables. Pearson’s chi-squared test for trend was used to compare the ordinal data. The Mann–Whitney U test was used to compare continuous variables. Kaplan–Meier estimator and log-rank tests were used to compare survival rates between groups. Cox proportional hazards regression was used to determine the univariate and multivariate hazard ratios (HRs) of the potential predictors of PFS and OS.

## Supplementary Information

Supplementary Information.
