# Supplementary material for: Circulating tumour cells at baseline and late phase of treatment provide prognostic value in breast cancer
Source: Sci Rep. 2021 Jun 29;11:13441. doi: 10.1038/s41598-021-92876-8 (PMC8241989; doi:10.1038/s41598-021-92876-8)
Supplement: Supplementary file 1 — Supplementary Information. [file 41598_2021_92876_MOESM1_ESM.pdf]

Supplementary information

Circulating tumour cells at baseline and late phase of treatment provide prognostic value in breast cancer

Shuyun Pang[1#], Hanjun Li[2#], Shu Xu[1, 4#], Liying Feng[1], Xueping Ma[1], Yanan Chu[1], Bingjie Zou[1*], Shaohua Wang[2*], Guohua Zhou[1, 3*]

[1] Department of Clinical Pharmacy, Jinling Hospital, State Key Laboratory of Analytical Chemistry for Life Science & Jiangsu Key Laboratory of Molecular Medicine, Medical School of Nanjing University, Nanjing, 210002, China

[2] Department of General Surgery, Jinling Hospital, Medical School of Nanjing University, Nanjing 210002, China

[3] School of Pharmaceutical Science, Southern Medical University, Guangzhou, 510515, China

[4] School of Basic Medical Science and Clinical Pharmacy, China Pharmaceutical University, Nanjing, 210000, China

# These authors contributed equally to this work.
*Corresponding Author:
Guohua Zhou, Tel/ Fax: (86) 25-80860195, E-mail: ghzhou@nju.edu.cn
Shaohua Wang, E-mail: wanglaifu2@126.com
Bingjie Zou, E-mail: zbj523@163.com

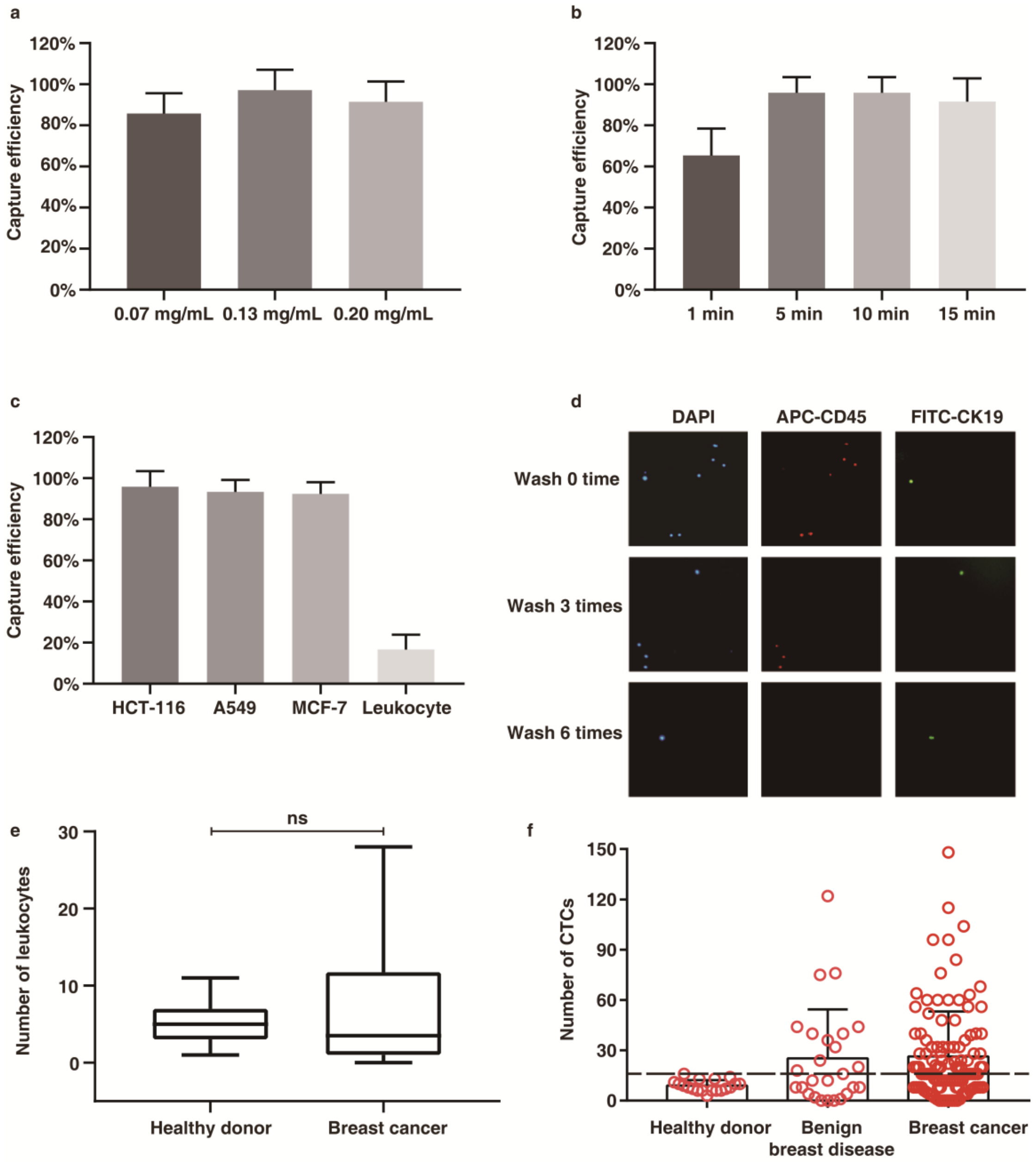

Figure S1. The performance of IMN-based CTC enrichment platform. (a) Capture efficiencies of MCF-7 cells using IMNs with different concentrations. (b) Capture efficiencies of MCF-7 cells by using IMNs with different incubation time. (c) Capture efficiencies of different epithelial cells using IMNs. (d) Stained captured cells with different number of wash times. (e) The number of leukocytes in healthy donors and patients with breast cancer. "ns" denotes non-statistical significance. (f) The number of CTCs, enriched by using IMNs, in healthy donors, patients with benign breast disease, and patients with breast cancer.

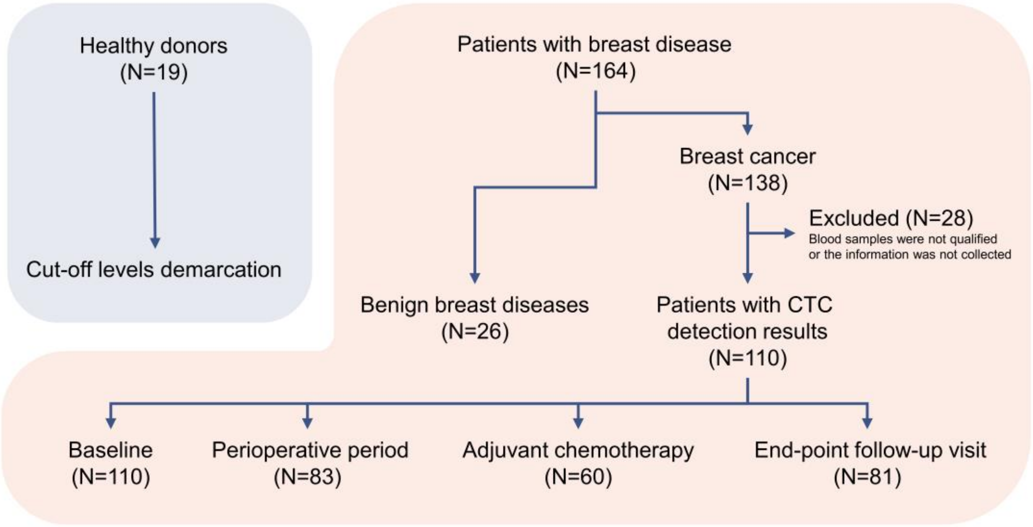

Figure S2. Flow Diagram.

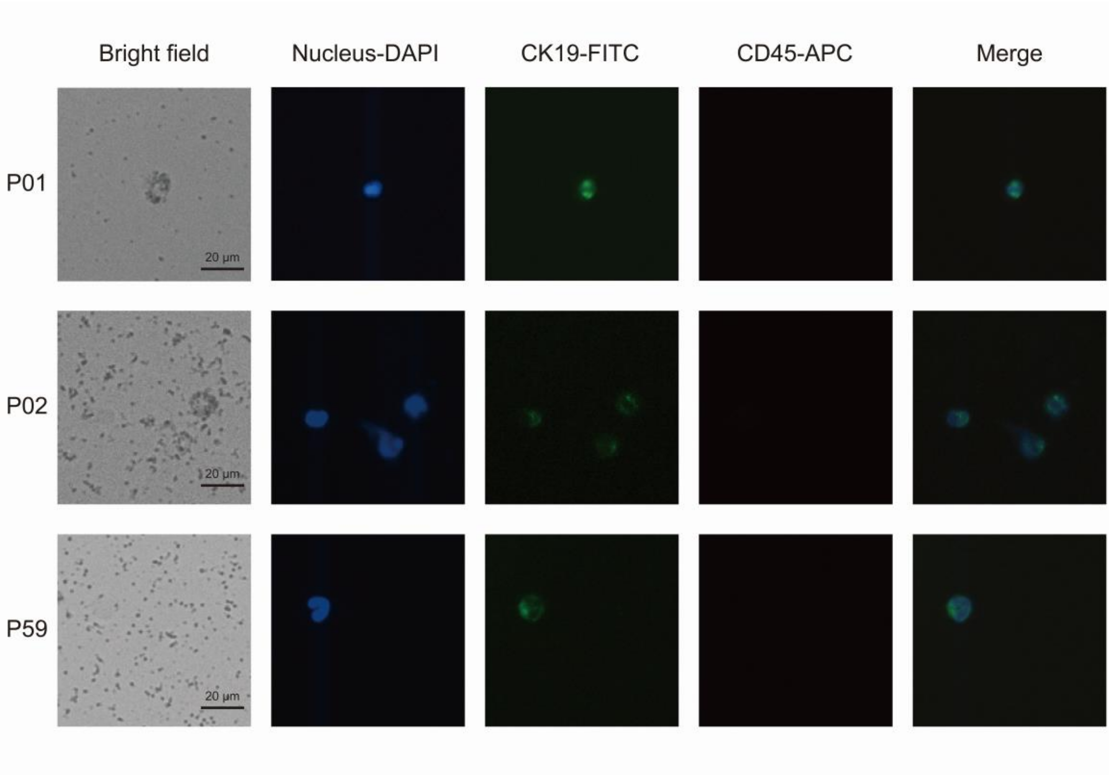

Figure S3. Representative images of CTCs found in 3 patients (P01, P02 and P59). CTCs are cytokeratin positive (green), CD45 negative (red), contains a DAPI nucleus (blue).

Table S1 CTC count of all enrolled patients

Patient ID	Baseline	Before surgery	1 d after surgery	7 d after surgery	14 d after surgery	Before initial chemotherapy	Before final chemotherapy	End-point follow-up visit
P01	20	15	14	/	13	“	8	“
P02	20	2	15	2	452	“	252	48
P03	7	24	4	/	0	“	4	0
P04	6	“	3	59	3	“	0	“
P05	39	“	107	/	26	“	20	“
P06	4	“	53	51	48	“	48	“
P07	1	“	71	/	58	“	16	“
P08	1	80	92	46	/	/	/	/
P09	20	“	6	/	5	“	26	“
P10	23	“	46	/	/	/	/	16
P11	29	“	47	/	/	/	/	/
P12	63	“	273	/	/	60	4	“
P13	15	“	/	/	/	/	/	/
P14	22	“	26	/	/	1	16	24
P15	2	“	11	/	/	/	/	/
P16	5	“	5	/	/	/	/	/
P17	12	/	/	/	/	/	/	/
P18	13	/	/	/	/	/	/	/
P19	115	/	/	/	/	/	/	84
P20	24	12	128	84	/	/	/	/

Table S1 CTC count of all enrolled patients (Continued)

Patient ID	Baseline	Before surgery	1 d after surgery	7 d after surgery	14 d after surgery	Before initial chemotherapy	Before final chemotherapy	End-point follow-up visit
P21	16	/	/	/	/	/	/	32
P22	28	28	64	/	/	/	/	/
P23	21	/	/	/	/	/	/	/
P24	104	16	120	/	/	/	/	48
P25	36	28	28	28	52	“	12	“
P26	16	20	24	96	8	“	80	“
P27	4	“	24	28	28	“	40	“
P28	16	12	24	28	4	“	4	“
P29	60	/	/	/	/	/	/	8
P30	148	16	16	0	0	“	20	“
P31	96	“	/	/	/	/	/	84
P32	20	/	/	/	/	/	/	24
P33	12	“	24	20	16	“	32	“
P34	52	20	4	/	8	“	16	“
P35	8	/	/	/	/	/	/	/
P36	32	“	/	/	/	/	/	/
P37	32	/	/	/	/	/	/	/
P38	8	“	8	16	/	4	4	“
P39	4	“	20	60	/	0	4	“
P40	8	“	28	/	/	0	12	“
P41	36	“	72	24	/	/	/	24
P42	12	“	24	/	/	/	/	12

Table S1 CTC count of all enrolled patients (Continued)

Patient ID	Baseline	Before surgery	1 d after surgery	7 d after surgery	14 d after surgery	Before initial chemotherapy	Before final chemotherapy	End-point follow-up visit
P43	12	80	16	0	/	/	/	0
P44	8	40	80	36	36	“	24	“
P45	8	8	8	16	/	/	/	16
P46	56	16	24	12	/	/	/	16
P47	16	“	36	4	/	/	/	4
P48	20	/	/	/	/	/	/	/
P49	96	“	92	24	/	/	/	16
P50	56	“	12	72	0	“	72	“
P51	40	“	52	28	28	“	0	“
P52	84	16	16	36	0	“	24	“
P53	16	“	8	/	12	“	48	“
P54	76	24	16	28	/	/	/	28
P55	12	“	24	24	/	/	/	16
P56	0	“	12	36	56	“	4	“
P57	16	20	4	20	20	“	16	“
P58	64	/	/	/	/	/	/	/
P59	8	64	100	/	16	“	8	“
P60	24	24	80	20	56	“	8	“
P61	0	“	72	20	12	“	0	“
P62	0	36	24	/	28	“	125	“
P63	16	/	/	/	/	/	/	8
P64	0	12	20	36	4	“	4	“

Table S1 CTC count of all enrolled patients (Continued)

Patient ID	Baseline	Before surgery	1 d after surgery	7 d after surgery	14 d after surgery	Before initial chemotherapy	Before final chemotherapy	End-point follow-up visit
P65	0	/	/	/	/	/	/	16
P66	16	/	/	/	/	/	/	12
P67	60	312	148	44	124	124	32	“
P68	8	4	16	/	/	/	/	12
P69	8	8	56	16	/	/	/	36
P70	24	“	40	12	/	/	/	12
P71	8	/	/	/	/	20	0	“
P72	24	/	/	/	/	12	16	“
P73	32	36	36	/	24	“	8	“
P74	20	/	/	/	/	16	44	“
P75	8	“	12	16	/	/	/	16
P76	12	“	52	52	25	“	32	“
P77	12	“	20	/	16	16	12	“
P78	12	0	8	4	/	/	/	8
P79	28	“	40	28	/	/	/	/
P80	0	“	20	12	12	“	8	“
P81	32	“	92	/	/	/	/	/
P82	60	/	/	/	/	/	/	/
P83	20	/	/	/	/	/	/	108
P84	20	12	76	/	48	“	44	“
P85	6	/	/	/	/	/	/	20
P86	40	8	24	/	88	“	0	“

Table S1 CTC count of all enrolled patients (Continued)

Patient ID	Baseline	Before surgery	1 d after surgery	7 d after surgery	14 d after surgery	Before initial chemotherapy	Before final chemotherapy	End-point follow-up visit
P87	12	“	16	4	44	“	4	“
P88	32	4	/	/	116	“	180	“
P89	4	“	/	24	20	“	20	“
P90	12	28	12	8	16	“	4	“
P91	60	/	/	/	/	60	60	“
P92	4	4	/	/	24	“	32	“
P93	68	/	/	/	/	68	28	“
P94	56	/	/	/	/	/	/	/
P95	48	/	/	/	/	/	/	/
P96	40	“	36	68	/	/	/	/
P97	0	8	28	/	32	“	16	“
P98	16	“	16	24	8	“	8	“
P99	8	12	28	12	12	“	24	“
P100	40	/	/	/	/	/	/	/
P101	0	40	8	/	8	“	32	“
P102	32	48	8	36	0	“	124	“
P103	20	“	44	12	/	/	/	/
P104	28	“	40	/	20	“	12	“
P105	8	0	32	28	44	“	16	“
P106	16	“	20	32	20	“	12	“
P107	20	/	/	/	/	16	36	“
P108	28	/	/	/	/	40	56	“

Table S1 CTC count of all enrolled patients (Continued)

Patient ID	Baseline	Before surgery	1 d after surgery	7 d after surgery	14 d after surgery	Before initial chemotherapy	Before final chemotherapy	End-point follow-up visit
P109	12	“	/	/	36	“	64	“
P110	48	76	72	/	/	/	/	/

Note:

1. “ indicate CTC count detected at this time point adopt the CTC count detected at previous time point. / indicate CTC detection did not proceed at this time point.

2. All CTCs were CK19-positive and EpCAM- positive.

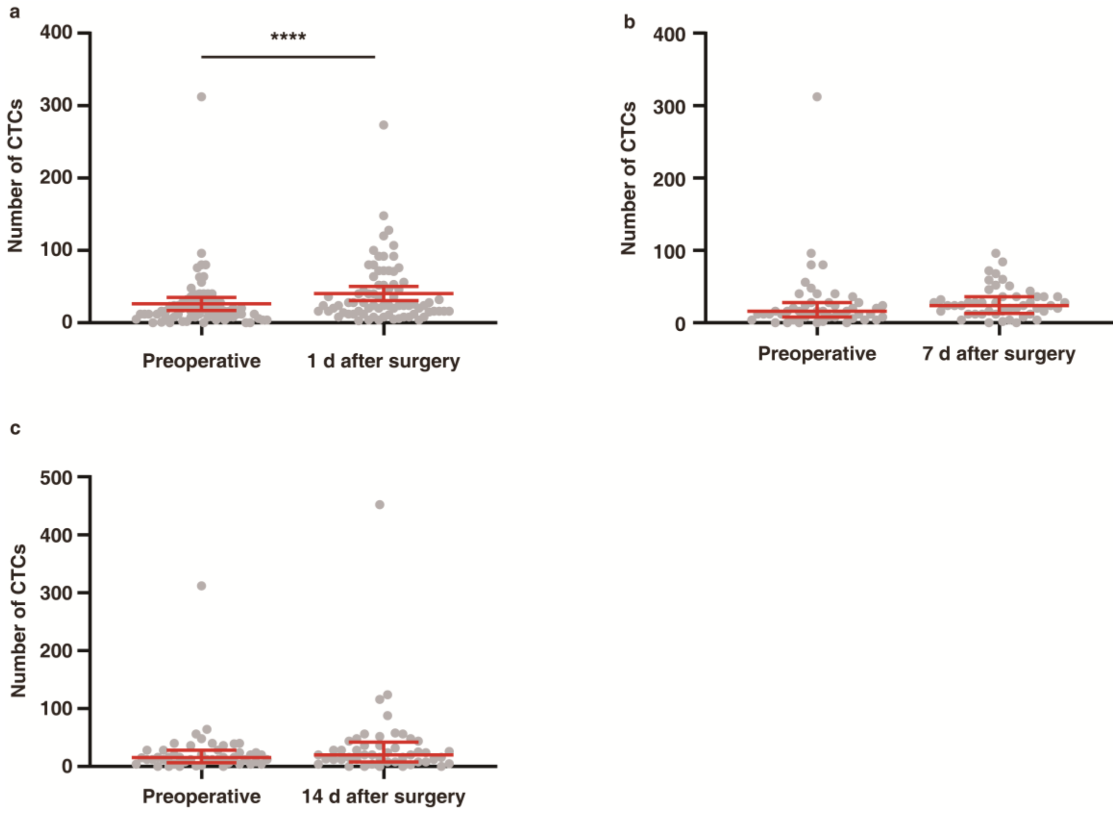

Figure S4. The comparison of CTC counts between preoperative and 1 d after surgery (a), preoperative and 7 d after surgery (b) and preoperative and 14 d after surgery (c). **** indicate $p \leq 0.0001$ 1 d after surgery versus preoperative.
